# Rajyoga meditation induces grey matter volume changes in regions that process reward and happiness

**DOI:** 10.1038/s41598-020-73221-x

**Published:** 2020-09-30

**Authors:** M. G. Ramesh Babu, Rajagopal Kadavigere, Prakashini Koteshwara, Brijesh Sathian, Kiranmai S. Rai

**Affiliations:** 1 Department of Physiology, Melaka Manipal Medical College, Manipal Academy of Higher Education [MAHE], Manipal, Karnataka 576104 India; 2 Department of Radiodiagnosis and Imaging, Kasturba Medical College, Manipal Academy of Higher Education, Manipal, Karnataka 576104 India; 3 Deputy Chair for Research, Geriatrics and Long Term Care Department, Rumailah Hospital, PO Box-3050, Doha, Qatar

**Keywords:** Cognitive neuroscience, Reward, Social behaviour

## Abstract

Studies provide evidence that practicing meditation enhances neural plasticity in reward processing areas of brain. No studies till date, provide evidence of such changes in Rajyoga meditation (RM) practitioners. The present study aimed to identify grey matter volume (GMV) changes in reward processing areas of brain and its association with happiness scores in RM practitioners compared to non-meditators. Structural MRI of selected participants matched for age, gender and handedness (n = 40/group) were analyzed using voxel-based morphometric method and Oxford Happiness Questionnaire (OHQ) scores were correlated. Significant increase in OHQ happiness scores were observed in RM practitioners compared to non-meditators. Whereas, a trend towards significance was observed in more experienced RM practitioners, on correlating OHQ scores with hours of meditation experience. Additionally, in RM practitioners, higher GMV were observed in reward processing centers—right superior frontal gyrus, left inferior orbitofrontal cortex (OFC) and bilateral precuneus. Multiple regression analysis showed significant association between OHQ scores of RM practitioners and reward processing regions right superior frontal gyrus, left middle OFC, right insula and left anterior cingulate cortex. Further, with increasing hours of RM practice, a significant positive association was observed in bilateral ventral pallidum. These findings indicate that RM practice enhances GMV in reward processing regions associated with happiness.

## Introduction

An important aspect of human life is the subjective experiences of happiness^1,2^. Happiness necessarily does not merely depend on wealth and material possessions^3,4^, but a kind of subjective feeling of positive emotions linked to pleasure and pleasant feelings. It is challenging to define happiness because of its subjective nature. It can be conceptualized by two general approaches, hedonic and eudaimonic. Hedonic approach importantly focusses on affective components such as positive emotions and pleasure, whereas the eudaimonic approach mainly focuses on personal well-being that includes concepts of personal growth, purpose in life and sense of autonomy. Individual happiness depends on the coherence functions of both approaches^5,6^. Happiness plays a crucial role in positive psychology and the fundamental goal of many individuals^7^, which can be achieved by practicing meditation regularly. Meditation awakens the individual’s inner consciousness and allows them to remain in conscious attention and positive state of mind despite fluctuating experiences of the external environment at any given moment. This helps to achieve happiness.

Studies on happiness by psychologists have helped to identify various features of happiness, whereas investigations by neuroscientists have identified the functional neuroanatomy of reward and pleasure together, providing a comprehensive insight into happiness^8^. Happiness is most relevant to reward and pleasure that are multifaceted psychological concepts. The process within the phenomenon of reward consists of motivation (wanting), learning and affect (happiness—pleasure liking)^5^. Physiological, pharmacological, and behavioral studies confirm that dopamine secreting regions in the nucleus accumbens (NAcc) located in the ventral striatum (VS) and ventral tegmental area (VTA) of the brain play a central role in reward^9^. The NAcc is known as ‘‘pleasure center’’ in the brain and is known to generate positive hedonic states regardless of any source of pleasure-inducing stimuli such as monetary reward, social reward, food, water, sex, drugs, etc. It involves the regulation of subjective and behavioral aspects of pleasure and reward. Further, activation of NAcc helps one to be in joyful states, achieved by repeated contemplative practices^10^. The dopamine secreting neurons in these regions play an essential role in positive emotions by mediating pleasure and positive reinforcement^11,12^. Whereas in a study by Pecina et al. 2006, opioid secreting neurons have been reported to be responsible for regulating pleasure in animals^13^. Though NAcc plays an important role in reward processing, it is also involved in modulating behavior to aversive and painful stimuli^14^.

Recent studies on reward centers emphasize that there are more extensive brain areas involved in the reward process apart from VTA and NAcc. Research studies on both animals and humans show that VTA is activated when reward is obtained. This center further innervates the NAcc, and the orbitofrontal cortex (OFC). Activation in medial OFC is associated with subjective reports of pleasure for musical, olfactory, and gustatory stimuli, at the time of a reward^15^. By using state of the art of neuro-imaging technologies like positron emission tomography and functional magnetic resonance imaging (MRI), various other regions, including cortical and subcortical regions, were identified to be involved in reward processing in the human brain. The cortical regions include OFC, cingulate cortex, medial prefrontal cortex (mPFC), and insula. The subcortical regions consist of the NAcc, ventral pallidum (VP), substantia nigra (SN), and VTA. These areas are also called “hedonic hotspots” which form reward-related circuitry in the brain^5,8^. These identifications reveal that the cortico-basal ganglia network is an integral part in reward circuit which is a central component for developing and monitoring motivated behavior. Besides, the limbic structure such as the hippocampus and amygdala send inputs to the VS and takes part in reward processing. VP plays a vital role in integrating the GABA, glutamate, opioid, and dopamine signals from NAcc, striatum, amygdala, and prefrontal cortex^9^. VP involves in learning, motivation, and reward through mesocorticolimbic circuits^16^. A study on SOHAM meditation suggests that higher grey matter density in VP is associated with reward processing and positive motivation^17^. Apart from these cortical and sub-cortical regions that involve in reward process, the precuneus also engages in different other processing such as reward-based decision-making task. It is a key structure in the default mode network that exhibits task-dependent connectivity within this network and right frontoparietal network^18,19^. Several studies reported the importance of precuneus, which has a role in the subjective experience of happiness, including emotional and cognitive components and subjective well-being^1,2,20,21^.

The term Raja yoga has been described in many scripts and texts including the Bhagavat Gita, and books rewritten by Yoga Sutra of Patanjali, Raja Yoga by Swami Vivekananda etc. In this manuscript Rajyoga meditation (RM) refers to Brahmakumaris school of thought which describes the basis for this type of meditation. RM is different from other types of meditation as it is an open-eye meditation technique. Most of the mediation techniques are closed-eye meditation practice focusing on their thoughts or count breathing, or chanting mantra, or remaining silent, etc. In RM practice, a person is instructed to realize himself/herself as self/soul (an eternal form of point of light) situated between the eyebrows while gazing on a meaningful external symbol (a point of light that is considered as symbolic representation of Supreme Soul/God)^22^. Also they are instructed to think positively about the innate qualities such as peace, love, bliss, etc. which are latent within the self^23,24^. There are very few studies published on RM, particularly in the field of neurocognitive research. A study by Sharma, K 2018, using electroencephalography (EEG), observed alpha and theta wave pattern in frontal and parietal areas of the brain that involves in the processes of regulation of selective and sustained attention for emotional and cognitive processing in long-term RM practitioners during meditation^23^. In another EEG study, enhanced lower alpha power in EEG recording was observed in the long-term as well as a short-term meditation practice. It suggests that the RM practitioners can move into a stable state of meditation within a few minutes irrespective of their meditation training. However, long-term practitioners can attain this state quicker than short-term meditators under varying external conditions. Hence RM practice, being an open-eye meditation technique, has potential applications in daily life^25^. A combined EEG and functional MRI study by Panda 2016, has provided the first evidence on the mental state during rest and meditative state of RM practitioners by analyzing on spatial extents and temporal dynamics of the default mode network^26^. These studies in RM practitioners suggest that the open-eye meditation technique plays a potential role in daily life and influences the brain regions that are mentioned in various other types of meditation techniques.

There are no studies have reported the impact of RM practice on inducing changes in reward processing areas of brain that are associated with subjective states of happiness. Therefore, we hypothesize that RM practice will induce grey matter volume (GMV) changes in brain regions that process reward and happiness and more years of RM experience will induce more GMV changes in brain regions that process reward. Thus, the aim of the present study was to identify RM induced neural changes in the brain regions that are involved in reward processing and its association with individual happiness scores measured by using Oxford happiness questionnaire (OHQ). To test our hypothesis, ROI masks created were used for the brain regions involved in reward process and happiness by applying threshold free cluster enhancement (TFCE) approach.

## Results

### Analysis of OHQ

**Figure 1:**
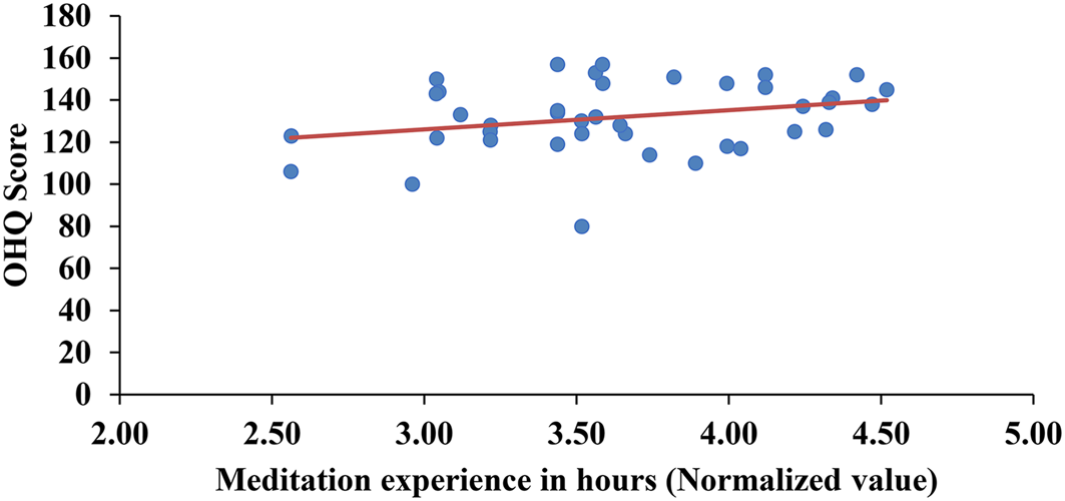
Correlation of OHQ scores with normalized hours of meditation experience showing a trend towards significance (*p* = 0.084).

RM practitioners showed a significantly higher OHQ score (mean ± SD: 131.88 ± 16.74), (*t* = 3.33, *df* = 78, *p* = 0.001) than non-meditators (NM) (mean ± SD, 118 ± 18.17). Further on analysis, a trend towards significant positive association was observed (Spearman’s correlation coefficient = 0.276, *p* = 0.084) when OHQ scores were correlated with normalized hours of meditation experience (Fig. 1).

### Comparison of GMV of reward centers of brain between RM practitioners and NM

**Figure 2:**
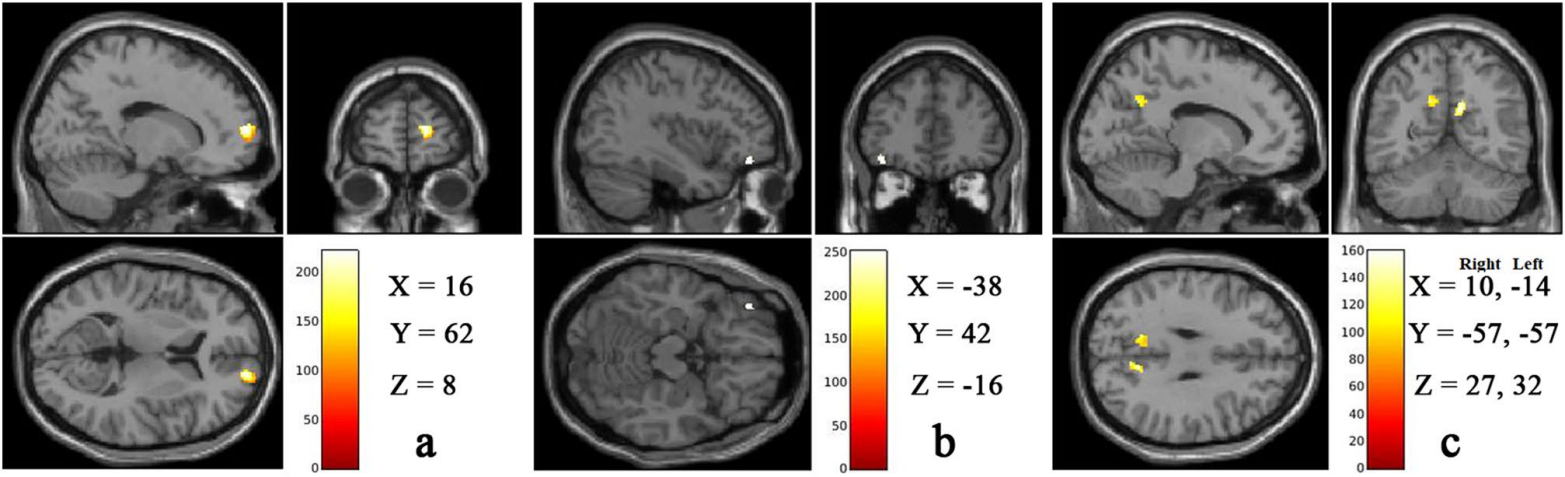
Representative structrual MRI images, showing significant difference (*p* < 0.05 FWE TFCE corrected) in (**a**) right superior frontal gyrus, (**b**) inferior orbitofrontal gyrus and (**c**) bilateral precuneus in RM practitioners than NM. Color bar represents TFCE-value. These representative structural MRI images were compiled and MNI coordinates were inserted into this compiled image using Adobe Photoshop version CS3 (https://www.adobe.com/in/).

In RM practitioners, a significant increase in GMV in the right superior frontal gyrus (SFG), left inferior OFC, and bilateral precuneus were observed as shown in Fig. 2. The details of peak voxel coordinates in MNI space, numbers of voxels in a cluster and p values are given in Table 1.

**Table 1:** GMV increase in brain regions that process reward and happiness of RM practitioners.

Brain regions	Side	MNI coordinates			k	*p*-value*
		X	Y	Z		
Superior frontal gyrus	Right	16	62	8	280	0.005
Inferior orbito frontal cortex	Left	− 38	42	− 16	45	0.037
Precuneus	Right	10	− 57	27	264	0.011
	Left	− 14	− 57	32	112	0.019

*p < 0.05 FWE TFCE corrected, k = cluster size (number of voxels).

### Association of GMV of brain regions that process reward and happiness with their OHQ scores in RM practitioners

**Figure 3:**
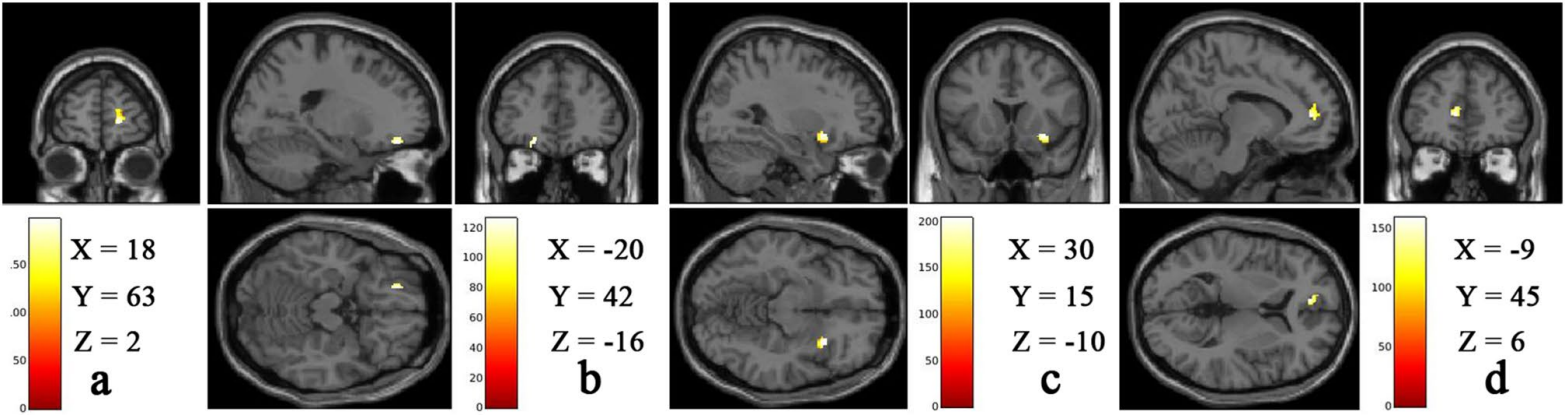
Representative structual MRI image, showing significant (*p* < 0.05, FWE TFCE corrected) positive correlation with GMV of (**a**) right superior frontal gyrus, (**b**) left middle orbitofrontal gyrus, (**c**) right insula and (**d**) left anterior cingulate cortex in RM practitioners. Color bar represents TFCE value. These representative structural MRI images were compiled and MNI coordinates were inserted into this compiled image using Adobe Photoshop version CS3 (https://www.adobe.com/in/).

The result of multiple regression analysis showed a correlation of OHQ scores with brain regions related to reward processing in RM practitioners and not in NM. A significant positive correlation was found in the right SFG, left middle OFC, right insula and left ACC (Fig. 3) in RM practitioners. No significant negative correlations in brain regions that process reward and happiness were observed in RM practitioners. Table 2 shows details of cluster size of the identified brain regions, x, y, z coordinates in MNI space and *p*-values.

**Table 2:** Positive association of OHQ scores with reward processing areas of the brain in RM practitioners.

Brain regions	Side	MNI coordinates			k	*p*-value*
		X	Y	Z		
Superior frontal gyrus	Right	18	63	2	357	0/008
Middle orbitofrontal cortex	Left	− 20	42	− 16	58	0.002
Insula	Right	30	15	− 10	155	0.001
Anterior cingulate cortex	Left	− 9	45	6	195	0.027

*FWE TFCE corrected *p* value, k = cluster size (number of voxels).

### GMV of brain regions that process reward and happiness associated with hours of meditation experience

**Figure 4:**
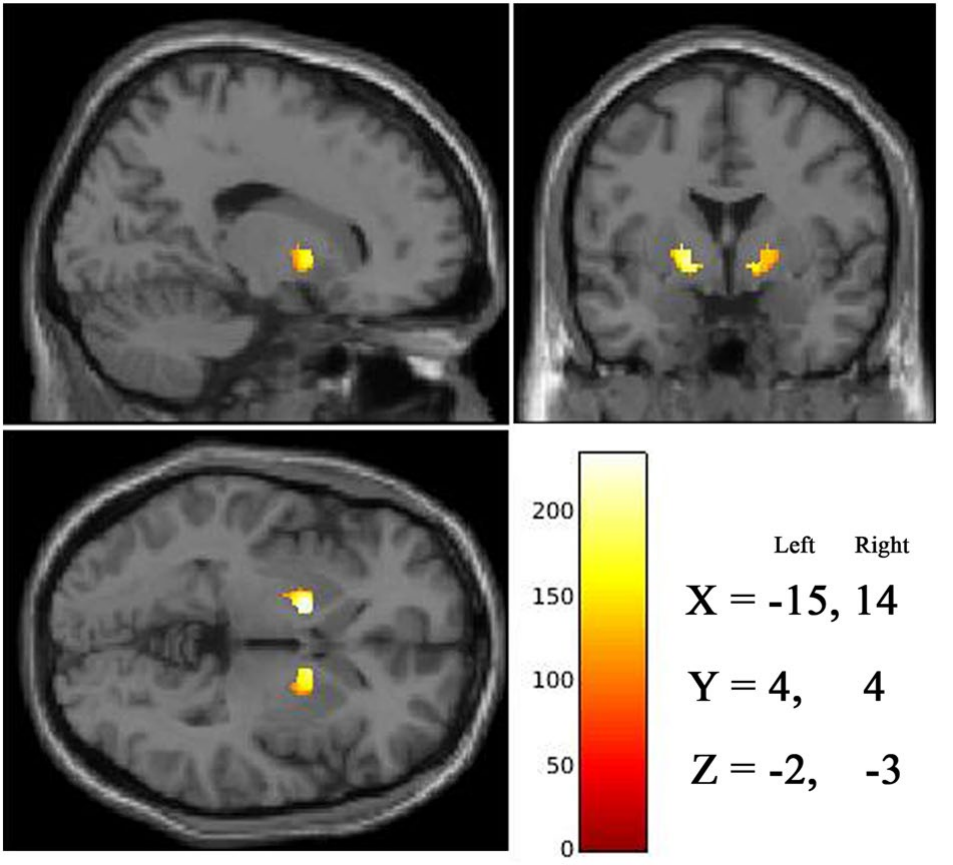
Representative structural MRI image, showing significant (*p* < 0.05 FWE TFCE corrected) positive association of GMV of bilateral ventral pallidum in RM practitioners with hours of meditation practice. Color bar represents TFCE value. These representative structural MRI images were compiled and MNI coordinates were inserted into this compiled image using Adobe Photoshop version CS3 (https://www.adobe.com/in/).

A significant positive association was found in bilateral VP of brain regions related to reward processing in RM practitioners on performing multiple regression analysis to correlate meditation experience, as shown in Fig. 4. There were no significant negative correlations observed in RM practitioners. Table 3 shows details of cluster size of the identified brain regions, x, y, z coordinates in MNI space and *p*-values.

**Table 3:** Correlation of meditation experience associated with GMV change in ventral pallidum in RM practitioners.

Brain regions	Side	MNI Coordinates			k	*p*-value*
		X	Y	Z		
Ventral Pallidum	Left	− 15	4	−2	220	0.009
	Right	14	4	− 3	203	0.016

*FWE TFCE corrected *p*-value, k = cluster size (number of voxels).

## Discussion

In the present study, RM practitioners had significantly higher OHQ scores than NM. Additionally, a trend towards significance in OHQ scores was observed in RM practitioners with more hours of RM experience. An earlier study on mindfulness meditation reported that happiness, self-compassion and higher levels of dispositional mindfulness are positively associated with frequency of meditation practice^27^. In contrary to this report, the present study shows that the association between happiness scores and RM experience was not significantly correlated, although there was a trend towards significance. This suggests that RM practice can improve happiness levels, irrespective of hours of RM experience. The higher happiness scores observed in experienced RM practitioners maybe because of emphasizing on a fundamental spiritual understanding of self and rediscovering the latent positive qualities within an individual^22,28^.

Further, group comparison between RM and NM showed that RM practitioners had higher GMV in the right precuneus. Studies show that this region along with the posterior cingulate cortex, is of primary importance since it receives the highest blood flow and cortical glucose metabolism at rest, as it is a part of the default mode network. Neuroimaging studies in humans reveal its essential role in subjective consciousness^29^ and subjective experience^30^ for integrating internal and external information^31^. Thus, the precuneus region appears to be involved in gathering information from different modes at a conscious level and is responsible for the subjective experience of happiness. A study on healthy volunteers using subjective happiness measure, purpose in life, positive and negative emotional intensity scales by Sato W 2015, reported a positive association between GMV of precuneus with subjective measures of happiness in healthy volunteers. This study suggests that the precuneus plays a vital role in mediating subjective experience of happiness by combining emotional and cognitive components of happiness^2^. Neuroimaging studies using VBM analytical technique on meditators have also shown findings of higher GMV in the precuneus region. In a pilot study by Kurth F 2014, observed higher GMV in the right precuneus after mindfulness meditation intervention and suggested that this region is essential for meditation practice^32^. This region has extensive communication with resting-state and external task regions. It processes self-relevant information which helps to engage in meditation^33,34^. Thus, in the present study, observation of increased GMV in precuneus in RM practitioners when compared with NM indicates that these changes in the precuneus may be due to higher subjective experiences of happiness in RM practitioners (indicated by their higher OHQ scores).

Moreover, the present study also shows significantly higher GMV in right SFG (part of mPFC) and left ACC that is significantly positively associated with OHQ scores in RM practitioners than those in NM. Both SFG and ACC are part of reward centers^35,36^. Studies on long-term Sahaja yoga meditation and others suggest that mPFC is responsible for maintaining meditation, attention, emotional regulation, behavior and cognitive control^37–39^. The interoceptive functioning of this region contributes to value-based decision-making. Such higher interoceptive abilities are associated with mindfulness meditation experience^40^. Positive thinking and happiness support brain growth, as well as the generation and reinforcement of new synapses, particularly in PFC, which serves as the integration center of all brain-mind functions^41^. The dorsolateral and medial PFC areas are involved in the choice of reward that varies over time and also important in normative decision-making related to monetary reward^42^.

Further, a study also shows that insight meditation experience is associated with cognitive control and self-regulation by dorsolateral PFC and dorsal ACC^43^. Greater activation in rostral ACC and dorsal mPFC in mindfulness meditators provides evidence that meditators can keep away from distractions and can control their emotions. The activity in the ACC decreases in long-term adept and Buddhist meditators, which suggests that, as these meditators become more efficient on focused attention, emotional regulation becomes unnecessary^44,45^. Integrative body-mind training, 30 min/day for five days, improves the cerebral blood flow to the ventral ACC, mPFC and insula which are the critical brain regions for self-regulation^46^. Research on transcendental meditation suggests that mPFC and ACC are essential in the mechanism of regulating mental state^47^. ACC is the complex structure that has an extensive connection with other reward regions such as VS and VP^9^. A positive hedonic environment attenuates the ACC activity and is responsible for behavioral adaptation^48^. Studies on ACC and amygdala in mindfulness meditators suggest that ACC helps to maintain the attention to a stimulus and the amygdala is responsible for generating emotions. Through these brain regions, meditation regulates negative thoughts and decreases emotional reactivity that helps to overcome psychiatric disorders such as depression and borderline personality disorders^49,50^. The higher GMV in right SFG and left ACC in the present study indicates that practicing RM may help to gain voluntary control over attention, behavior and emotional regulation.

Additionally, in the present study, a significant increase in GMV in left inferior OFC, right insular cortex and a smaller left middle OFC that was positively associated with happiness scores were observed in RM practitioners. Anatomically, insular region has bidirectional connections with the structures that are related to reward and decision making which includes the OFC, ACC, NAcc, and the amygdala^51^. Insular cortex plays a key role in integrating information related to emotions and introspection. These information are then forwarded to the OFC and ACC, which influence decision making^52^. Functional MRI study in several Buddhist traditions including Shamatha/breath-focus, Vipasana/insight, compassion, Tong-Len and other Tibetan style meditations, suggests that the individuals with more meditation experience may have an enhanced awareness of present moment experience^53^. Insula, along with ACC and striatum, are involved in the maintenance of meditative states with less effort and attentional control^54^. OFC regulates the process of reward and punishment^55^. Increased activation of this region has been observed during reward or loss incurred^56^. A study by Estela C et al. 2009, reported a graded increase in the activation of this region in relation to reward^52^. Greater grey matter concentration in the medial OFC is dependent on mindfulness meditation training might reflect an improved ability to modify emotional responses^57^. Thus, these supporting findings indicate that long-term RM practice may enhance awareness of present moment experience, attentional control and helps the practitioner to maintain the meditative states with less effort.

Finally, the interesting finding in the present study is the significant correlation of GMV in bilateral VP with hours of RM experience. VP is part of the basal ganglia and is well known to play an important role in the planning and regulation of motor activity. Modern-day high-resolution human brain imaging technology validates and clarifies the role of the VP in human emotional response and psychiatric disorders^58^. Accumulating evidence from recent studies reveals that apart from its role in motor activity, the VP also plays an essential role in hedonic response to a pleasant stimulus. Also, VP produces this response in the background activity of ACC^5,59^. The dopaminergic fibers regulate the activity of glutamatergic neurons from the frontal cortex to striatum and project back to the frontal cortex via pallidum and thalamus. It is reported that increased dopamine release during meditation, which suppresses the cortico-striatal glutamatergic transmission, is associated with motor control. The increase of dopamine associated with meditation training may contribute to dopaminergic neurogenesis^37^. VP is one of the central components of the reward circuit which sub-serve the functions of positive motivation and reward^9^. The VP receives a reward signal from NAcc and other forebrain limbic structures to execute it^60,61^. Studies on volunteers who had chronic pain and sadness correlate with the deactivation of m-opioid receptors in the VP. This is identified that they had behavioral problems associated with negative affect which suggests that the m-opioid receptors in the VP regulate the positive affect. It regulates the affective/motivational process at the conscious or unconscious level^58,62,63^. A study on SOHAM meditators suggests that higher grey matter density in VP is associated with reward and positive motivation^17^.

Additionally, an earlier study shows that reward-centers down-regulate hedonic effects themselves as a result of repeated exposure of worldly rewards corresponding to the conditioned suppression of dopamine^64^. Everyday thought patterns that are repetitive, predictable and rewarded behaviors initially support their development but later may lose their ability to process the rewarded behavior which has become repetitive. This may be due to a continuous suppression of dopamine-related activity in NAcc^10^ which is the key region in reward processing. This may explain the reason for not findings a significant increase in GMV or any correlation in VS and NAcc, in the present study. Moreover, the interesting finding in the present study is the significant positive association of GMV in bilateral VP with RM experience, which may indicate that RM practice may contribute to the automatic mind states of developing positive motivation and experience of reward.

The potential limitation in the present study is the education levels which were not matched between groups. Education plays an important role in brain modulation based on the individual’s education levels and may introduce a confounding effect if not matched^65–67^. The hippocampus and amygdala regions play an important role in the reward processing^9^ are directly or indirectly influenced by levels of education of the participants. In this study, no significant changes were observed in the hippocampus and amygdala either in *t*-test or in correlation analysis even after adding education levels as a covariate. In the future, repeating a similar type of study with larger sample size and matching years or levels of education may help to overcome this confounding effect.

## Conclusion

This study provides the first evidence for the underlying neural substrate of higher GMV changes in reward processing areas of RM practitioners including right SFG, left ACC, left OFC, right insula and bilateral VP as well as precuneus an important structure that mediates happiness. This was observed to be associated with higher happiness scores in RM practitioners compared to the happiness levels in NM. Besides, years of RM experience was observed to enhance GMV in these reward processing regions to greater extents. However, the happiness scores were not positively associated with hours of meditation experience, indicating that RM practice provides happiness, irrespective of the time span of RM experience, and RM practice may contribute to the automatic mind states of developing positive motivation and experience of reward.

## Methods

### Participants

The total number of RM practitioners and non-meditators (NM) in both the groups were eighty (n = 40/group). They were matched for age, gender and handedness. Subjective happiness levels were measured using OHQ and MRI obtained was used to study structural changes in the brain regions that process reward and happiness. The social-demographic details of all participants are given in Table 4. The participants who had the following exclusion criteria for the study, such as metabolic disorders, addiction to drugs or alcohol, mental disorders, physical illness, and claustrophobia, were not selected. Participants who were practicing only RM and regularly attending spiritual and meditation classes were recruited from Brahma Kumaris Rajyoga Meditation Centre, Manipal, Udupi, and Mangalore cities of Karnataka, India. The hours of meditation experience were calculated by their years of meditation experience, hour/s spent in meditation per day and hours spent in meditation retreat. NM participants were volunteers for this study and were selected from Manipal, Udupi regions of Karnataka, India. After obtaining approval from the institutional ethical committee (IEC 566/2013) Kasturba Hospital, MAHE, Manipal for human studies, this study was conducted. Standard ethical guidelines laid down by Declaration of Helsinki-World Health Organization was followed for all methods in this study. All the participants were informed about the study well in advance and MRI safety precautions were explained to all. Before the study, a signed-in informed consent form was obtained from each participant.

**Table 4:** Socio-demographic details of participants.

	Meditators (n-40)	Non-meditators (n = 40)	P value
Age (mean ± SD) (Range: minimum–maximum)	41.28 ± 8.05 (26.4–52.85)	40.06 ± 7.21 (26.32–55.16)	0.48 (t-test)
**Gender (n)**			
Male	20	20	1.0 (X^2^ test)
Female	20	20	
Handedness (n)			
Right	39	39	1.0 (Fisher’s exact test)
Left	1	1	
Educational levels			
School	20	21	0.001 (Fisher’s exact test)
Degree	3	14	
Postgraduate	13	5	
PhD/PDF	4	0	
Meditation Experience (in hours)	8022.24 ± 8767.24	NA	NA

*NA* not applicable.

### Psychological test tool

OHQ, developed by Peter Hills in 2002^68^, a self-reported cognitive scale that measures subjective happiness level was used for this study. Psychologists and sociologists widely use it as a tool for assessing happiness status^69–73^. OHQ is a unidimensional scale with 29-items, designed to measure happiness status, which is a reliable and valid questionnaire with reliability alpha coefficient of 0.91. It employs a 6-point Likert-type format of the response, from ‘strongly disagree’ to ‘strongly agree, to measure self-reported happiness score. Higher the score more will be the happiness level. The filled-in OHQs by all participants were collected and the total score for the 29 items were calculated. In this study, the overall score of the OHQ was considered.

### MRI acquisition

Structural MRI data were acquired for all participants with Philips Acheiva Medical Imaging 1.5 T scanner with 8 SENSE head-coil. High-resolution three-dimensional sets of data of the whole brain were collected using T1 weighted, TFE—turbo field echo sequence. It consists of sagittal partitions with slice thickness 1 mm, TE/TR (echo/repetition time) 3.703 ms /7 ms, flip angle 8°, matrix 256 × 256, 1 × 1 voxel dimension, and field of view 256 mm which yielded 175 slices encompassing the whole brain.

### Image analysis

Voxel based morphometric analysis is a method for investigating neuroanatomical alterations in the brain in an unbiased and objective way using T1-weighted structural MRI scans. VBM analysis involves the measurement of voxels in the brain MRI, and regional GMV can be obtained^74^. Statistical comparison of GMV between two or more different experimental groups can be analysed and the statistical significant differences in the volume of brain regions can be established. In recent years, this technique has become very popular and it is also used to detect stimuli-induced morphological changes in the brain where functional MRI is not suitable for study^75^. In the present study to detect GMV changes in brain, VBM method was applied by using the CAT12 toolbox version 12.6 (https://www.neuro.uni-jena.de/cat/) installed in the SPM12 software toolbox (ftp://ftp.fil.ion.ucl.ac.uk/spm), and the common platform for this software was MATLAB software version R2019a.

Initially, all the DICOM images were converted into single file NIFTY format by using MRI Convert version 2.1.0 (https://lcni.uoregon.edu/downloads/mriconvert). Then all the converted images were manually reoriented and fixed with the anterior commissure as origin which matches the canonical image template provided in SPM12. All the preprocessing steps for VBM analysis were carried out using the CAT12 tool as mentioned in the VBM manual (https://www.neuro.uni-jena.de/cat12/CAT12-Manual.pdf). For initial segmentation, a sensitive segmentation method than the normal VBM method known as DARTEL (Diffeomorphic anatomic registration through exponentiated Lie algebra algorithm) segmentation method was applied to segment the MRI images. By this method, we obtained affine transformed images of grey and white matter. TIV estimate option in CAT12 tool generates total intracranial volume (TIV) for each subject and was used for homogeneity check and as nuisance covariate. After the segmentation process, all images were inspected manually by displaying one slice for all images. Moreover, quality of data such as resolution, noise and bias were also obtained. Using this information, the weighted average quality of all images were B or higher, which represents very good quality. Therefore, none of the images were excluded from the analysis. A study-specific template was created representing the sample of study using affine registered grey and white matter images. Further, this template was used to generate normalized, modulated, non-linear DARTEL warped segmented images of grey and white matter in Montreal Neurological Institute (MNI) Space. A FWHM (full width at half maximum) isotropic Gaussian kernel with 8 mm size was used for VBM statistical analysis.

### Creating Region of Interest (ROI) Masks

A 3D ROI sphere mask was created for the regions that represent the reward processing regions using WFU-Pickatlas (Wake Forest University) atlas tool (https://fmri.wfubmc.edu/software/pickatlas) to test the study hypothesis. Bilateral union masks for cortical and sub-cortical centers using “x, y, z” coordinates in MNI space mentioned in the studies related to reward and meditation are as follows: for cortical centers 8 mm masks created for OFC (± 27, 42, − 14)^15,76,77^, ACC (± 6, 51, 10)^78^, insula (± 30, 10 -15)^38^, and precuneus (± 6, 54, 22)^77^, regions. For sub-cortical structures, 4 mm masks were created for NAcc (± 10, 9, − 4)^15^, VP (± 14, − 10, − 8)^59^, basolateral amygdala (± 32, 0, -26)^79^, and hippocampus (± 18, − 37, − 11)^80^ For mid-brain regions that involve in reward processing, 2 mm masks were created including SN (± 12, − 12, − 12 ), and VTA (0, − 15, − 12) regions^15,81^. Totally 11 bilateral union masks for reward centers were created. Since this tool creates masks with voxel dimensions of 2 mm in x, y, z directions by default, using a co-register (reslice) option in SPM12, all the masks were co-registered with a VBM output image to get the same voxel dimensions for all ROI masks created.

### Statistical analysis

SPSS version 22, was used to perform *t*-test and find out the difference in mean OHQ score between groups. A correlation analysis was performed to correlate hours of meditation experience with OHQ score in RM. Since hours of meditation experience were not normally distributed (mean ± SD: 8022.24 ± 8767.69), a normalized value for hours of meditation experience (mean ± SD: 3.64 ± 0.52) and Spearman correlation coefficient was considered in the correlation analysis. Correlation results were reported significant if the *p*-value < 0.05.

For VBM analysis, to compare between 2 groups, the basic statistical model was applied to conduct a *t*-test to find out voxel-wise gray matter (GM), and white matter (WM) volume changes between NM and RM using the CAT12 toolbox. ANCOVA was performed with a flexible factorial design to compare group differences and adjusted for TIV, age, and education levels which were entered as covariates to remove confounding effect due to variable head/brain size, aging process and influence of education. An absolute threshold masking 0.1 was applied to all the smoothed images to avoid edge effect around the borders of GM and WM. The t-contrasts [-1 1] for NM > RM and [-1 1] for RM > NM were used to measure group differences. A multiple regression analysis was performed to correlate the reward center of the brain with hours of meditation experience and OHQ by entering these variables as independent variables. Since there was collinearity (*cos θ* = 0.7) between these two variables which was identified from the SPM design orthogonality output map. Hence, multiple regression analysis was performed separately for these two independent variables to avoid multi collinearity issues. For both multiple regression analyses TIV, age, and education level were entered as nuisance covariates. The *t*-contrasts [1] for positive correlation and [− 1] for negative correlation were used. All other parameters were kept the same as for the *t*-test. TFCE, a non-parametric methods, with 5000 permutations^82^ was applied by using the TFCE tool version 185 (https://dbm.neuro.uni-jena.de/tfce/). This approach combines focal effects with large voxel height and size of the cluster in VBM data. It is more reliable and effective for group-level analysis with or without the requirement of high and uniform spatial smoothness^83^. The 3D ROI sphere masks were used for small volume correction in the TFCE approach. The resultant voxels were considered significant, if it is above the threshold value *p* < 0.05, after correcting for family wise error (FWE) using multiple comparison with TFCE in the ROIs. The final output, SPM statistical map image, was overlaid on the single subject representative image from SPM canonical template.
